# Interactions Between Potentially Toxic Nanoparticles (Cu, CuO, ZnO, and TiO_2_) and the Cyanobacterium *Arthrospira platensis*: Biological Adaptations to Xenobiotics

**DOI:** 10.3390/nano15010046

**Published:** 2024-12-30

**Authors:** Ludmila Rudi, Liliana Cepoi, Tatiana Chiriac, Svetlana Djur

**Affiliations:** Institute of Microbiology and Biotechnology, Technical University of Moldova, MD 2028 Chisinau, Moldova; liliana.cepoi@imb.utm.md (L.C.); tatiana.chiriac@imb.utm.md (T.C.); svetlana.djur@imb.utm.md (S.D.)

**Keywords:** cyanobacterium *Arthrospira platensis*, nanoparticles, relation, adaptive mechanism, oxidative stress, potential toxicity

## Abstract

(1) Background: The widespread use of nanoparticles (NPs) implies their inevitable contact with living organisms, including aquatic microorganisms, making it essential to understand the effects and consequences of this interaction. Understanding the adaptive responses and biochemical changes in microalgae and cyanobacteria under NP-induced stress is essential for developing biotechnological strategies that optimize biomolecule production while minimizing potential toxicity. This study aimed to evaluate the interactions between various potentially toxic nanoparticles and the cyanobacterial strain *Arthrospira platensis*, focusing on the biological adaptations and biochemical mechanisms that enable the organism to withstand xenobiotic exposure. (2) Methods: The cyanobacterium *Arthrospira platensis* CNMN-CB-02 was cultivated under optimal laboratory conditions in the presence of CuNPs, CuONPs, ZnONPs, and TiO_2_NPs. Biochemical analyses were performed on the collected biomass. (3) Results: Various interactions between nanoparticles (NPs) and the cyanobacterial culture were identified, ranging from hormetic effects at low concentrations to evident toxic effects at high concentrations. NP toxicity was observed through the reduction in photosynthetic pigments and the disappearance of phycobiliproteins. Notably, NP toxicity was not always accompanied by increased malondialdehyde (MDA) levels. (4) Conclusions: *Arthrospira platensis* exhibits unique adaptive mechanisms under NP-induced stress, offering the potential for controlled NP applications in biotechnology. Future research should further explore the relationship between nanoparticle types and cyanobacterial responses to optimize biomolecule production.

## 1. Introduction

Research on microalgae and cyanobacteria in relation to nanoparticles represents a significant area of study with numerous purposes and profound biotechnological implications [1,2]. These autotrophic organisms, which use sunlight, carbon dioxide, and nutrients for growth, are distinguished for their high growth rates and adaptability to diverse environmental conditions. In many cases, microalgae can assimilate metals in the form of nanoparticles rather than their ionic forms, a mechanism that unlocks unique opportunities for biotechnological applications [1,3,4]. Nanoparticles can act as stimulators of various biosynthetic processes. Well-known methods exist for stimulating lipid synthesis in microalgal biomass for biofuel production [5]. Microalgae such as *Trachydiscus minutus* and *Pavlova lutheri* are mentioned as lipid producers that have been exposed to zero-valent iron nanoparticles. It is believed that the stimulatory effect of nanoparticles results from adaptation to the oxidative stress induced by these particles [6]. Understanding the adaptive mechanisms of microalgae and cyanobacteria is essential, as it enables the controlled use of nanoparticles to stimulate the biosynthesis of pigments, antioxidants, lipids, and other bioactive compounds, which have valuable applications in the pharmaceutical, food, and cosmetic industries [7]. Such research contributes to the development of sustainable technologies that harness the ability of microalgae to convert toxic substances into beneficial products [2]. Furthermore, this field provides deeper insights into the molecular and physiological mechanisms by which these organisms enhance their resistance to nanoparticle-induced stress [8].

The cyanobacterium *Arthrospira platensis* (spirulina) is renowned for its nutritional value and bioactive compounds, which have diverse applications in medicine, the pharmaceutical industry, and nutrition [9,10,11,12]. With its simple cellular structure, this organism produces components such as proteins, polysaccharides, phycobiliproteins, and lipids that contain gamma-linolenic acid (omega-6), which plays a crucial role in antioxidant protection mechanisms. These compounds also facilitate complex interactions with nanoparticles. Its ability to rapidly adapt to external factors makes spirulina a suitable model for investigating both the toxic effects and the tolerance potential of microalgae and cyanobacteria toward xenobiotics in nanoparticle form [13]. Spirulina is particularly well known for its resistance to various stress factors, including those induced by heavy metals and other toxic particles [14]. This resilience allows for the study of nanoparticle toxicity in a relatively robust model, enabling the identification of nanoparticle concentrations and types that induce adaptive responses or cellular detoxification mechanisms [15,16]. As an important marker in monitoring oxidative stress intensity and therefore toxic effects, malondialdehyde (MDA) is used to measure lipid peroxidation levels. The increase in MDA concentrations following nanoparticle exposure suggests damage to cell membranes and other internal structures due to the free radicals they generate. Thus, measuring MDA can provide essential information about the toxic impact of these particles on microalgae and the cellular protection mechanisms developed to cope with oxidative stress [17].

Nanoparticles such as copper (CuNPs), copper oxide (CuONPs), zinc oxide (ZnONPs), and titanium dioxide (TiO_2_NPs) have a wide range of applications, from fungicides and bactericides in agriculture to active ingredients in cosmetics and pharmaceuticals [18,19,20,21]. These nanoparticles can generate reactive radicals that induce oxidative stress, damaging cellular structures [22,23,24]. Specifically, CuNPs, CuONPs, and ZnONPs release metal ions that interact with proteins, while TiO_2_NPs disrupt photosynthesis by reducing light availability. The response of microalgae to these nanoparticles is diverse, though there are some common aspects [12,25,26,27].

Due to their extremely small size and unique properties, the interaction of nanoparticles with organisms at the molecular and cellular levels opens up opportunities to explore new adaptation and survival mechanisms in microalgae and cyanobacteria. From a biotechnology perspective, two aspects of the interaction between nanoparticles (NPs) and living organisms, including photosynthetic microorganisms, emerge: the stimulatory effects on certain processes which can be applied in biotechnology on the one hand, and the toxic effects which can compromise cell viability as well as the quality of the products obtained from them on the other hand. This study aims to evaluate the interaction between several types of potentially toxic nanoparticles—Cu, CuO, ZnO, and TiO_2_—and the cyanobacterium *Arthrospira platensis*, as well as to identify the biological adaptations developed to withstand exposure to xenobiotics.

## 2. Materials and Methods

### 2.1. The Cyanobacterial Strain Arthrospira platensis, Mineral Media, and Experimental Conditions

The cyanobacterium *Arthrospira platensis* CNMN–CB–02 (spirulina) strain obtained from the National Collection of Non-Pathogenic Microorganisms, the Institute of Microbiology and Biotechnology, the Technical University of Moldova, was used in this study. The strain was cultivated for 6 days in a mineral medium with the following composition (Sigma-Aldrich, Merck KGaA, Darmstadt, Germany): NaNO_3_—2.5 g/L; NaHCO_3_—8.0 g/L; NaCl—1.0 g/L; K_2_SO_4_—1.0 g/L; Na_2_HPO_4_—0.2 g/L; MgSO_4_·7H_2_O—0.2 g/L; H_3_BO_3_—2.86 mg/L; MnCl_2_·4H_2_O—1.81 mg/L; CuSO_4_·5H_2_O—0.08 mg/L; MoO_3_—0.015 mg/L; FeEDTA—1 mL/L. The cultures were grown in 250 mL Erlenmeyer flasks containing 100 mL of culture medium at a temperature of 28 ± 1 °C, pH 8–10, and illumination intensity of 37–55 µmol photons/m^2^·s with periodic agitation. On the first day of cultivation, nanoparticles were added to the culture medium at concentrations ranging from 0.01 to 30 mg/L.

### 2.2. Nanoparticles

The following types of nanoparticles were used in this study:

Copper nanoparticles (CuNPs): product code 774081 (Sigma-Aldrich, Merck KGaA, Darmstadt, Germany), with an average particle size of 25 nm (TEM) and a purity of 99.5%.

Copper oxide nanopowder (CuONPs): product code 544868 (Sigma-Aldrich, Merck KGaA, Darmstadt, Germany), with a particle size of 50 nm (TEM).

Zinc oxide nanoparticles (ZnONPs): product code 721077 (Sigma-Aldrich, Merck KGaA, Darmstadt, Germany), with particle sizes < 100 nm (TEM). Titanium dioxide nanoparticles (TiO_2_NPs): product code 718467 (Sigma-Aldrich, Merck KGaA, Darmstadt, Germany), with a size of 21 nm (TEM) and a purity of ≥99.5%.

To disperse Cu, CuO, and TiO_2_ nanoparticles and prevent the formation of aggregates, nanoparticle solutions in deionized water were subjected to ultrasonication at a frequency of 22 kHz and an ultrasonic intensity of 7 W/cm^2^ for 5 min, with agitation intervals of 10 s followed by 30 s of rest.

### 2.3. Quantitative Biomass Determination

Quantitative biomass determination was performed using an indirect spectrophotometric method. At the end of the cultivation period, the absorbance of the *Arthrospira platensis* suspension was measured at 680 nm (Spectrophotometer T80 UV/VIS, PG Instruments, Alma Park, Leicestershire, UK). Biomass quantification, expressed in gravimetric units (g/L), was achieved through a calibration curve that describes the correlation between suspension absorbance and cell mass. To ensure the accuracy of the spectrophotometric measurements, the *A. platensis* suspension was subjected to serial dilutions to maintain absorbance values within the range of 0.1–0.4, where a linear relationship between viable biomass concentration and absorbance is observed.

### 2.4. Biochemical Analysis

The reagents used for the biochemical tests are products of Sigma-Aldrich, Merck KGaA, Darmstadt, Germany.

#### 2.4.1. Sample Preparation for Biochemical Analysis

For biochemical analysis, the biomass of *Arthrospira platensis* (control and experimental samples) was separated from the culture medium by filtration, demineralized with 2% ammonium acetate solution, and washed with double-distilled water to remove residual salts. The biomass was then resuspended in distilled water and standardized to a concentration of 10 mg/mL. The biomass samples underwent repeated freeze–thaw cycles to release intracellular components and homogenize the suspension (six consecutive cycles of freezing at −20 °C and thawing at room temperature). The prepared samples were stored at −20 °C.

#### 2.4.2. Protein Determination

Protein content was determined using the Lowry method, which involves forming a copper–protein complex in an alkaline medium [28]. Proteins were extracted by treating 10 mg of biomass with 0.9 mL of 0.1 N NaOH solution for 30 min. A 0.2 mL aliquot was taken from the resulting hydrolysate and diluted with 0.8 mL of distilled water. To the diluted solution, 1.5 mL of a complex reagent was added, prepared by mixing 2% Na_2_CO_3_ in 0.1 N NaOH and 0.5% CuSO_4_ in 1% sodium tartrate in a volumetric ratio of 49:1. After a 10-min reaction period at room temperature, 0.5 mL of Folin–Ciocalteu reagent diluted at 1:3 was added. At the end of a 40-min reaction period, the absorbance of the solution was measured at a wavelength of 720 nm. Protein concentration was calculated using a calibration curve with bovine serum albumin as the reference standard.

#### 2.4.3. Phycobiliprotein Determination

Phycobiliprotein content was determined spectrophotometrically from aqueous extracts obtained from the biomass of *Arthrospira platensis*. A 1.0 mL sample of biomass, standardized to 10 mg/mL and subjected to repeated freeze–thaw cycles, was centrifuged at 11,000 rpm for 3 min (Hettich centrifuge MIKRO 22R, Andreas Hettich GmbH & Co. KG, Tuttlingen, Germany). The absorbance of the extracts was recorded at wavelengths of 620 nm and 650 nm. Quantitative calculation of phycobiliprotein content was performed using the following equations [29]:

Phycocyanin (PC) (mg/mL) = (Abs_620_ − (Abs_650_ × 0.7))/7.3

Allophycocyanin (APC) (mg/mL) = (Abs_650_ − (Abs_620_ × 0.208))/5.09

Phycobiliprotein content was expressed as a percentage relative to the dry biomass weight.

#### 2.4.4. Chlorophyll and Total Carotenoid Determination

The chlorophyll a and total carotenoid content were determined spectrophotometrically using a method based on pigment extraction in ethanol solvent. The experimental procedure involved suspending 10 mg of biomass in 1 mL of 96% ethanol, followed by pigment extraction through continuous stirring for 12 h at room temperature. The samples were then centrifuged at 5000 rpm for 10 min. Pigment quantification was performed by measuring the absorbance at 450 nm, 649 nm, and 665 nm wavelengths. The concentrations of pigments were calculated using the equations proposed by Lichtenthaler (1987) [30]:

Chlorophyll a (mg/mL) = (13.36 × Abs_665_ – 5.19 × Abs_649_)/1000

Total carotenoids (mg/mL) = (1000 × A450 – 2.05 × Chlorophyll a)/221

The pigment content was expressed as a percentage of dry biomass.

#### 2.4.5. Carbohydrate Determination

Carbohydrate content was determined using a spectrophotometric method based on the specific reaction with Anthrone reagent in an acidic medium. This method measures the intensity of the hydroxymethylfurfural coloration formed, which is proportional to the carbohydrate concentration (detection range: 0.02–0.10 µg/mL). The experimental protocol involved adding 20 µL of biomass suspension (10 mg/mL) to 2 mL of 0.5% Anthrone reagent in 66% sulfuric acid. Hydrolysis was conducted in a boiling water bath (Gesellschaft für Labortechnik mbH, Burgwedel, Germany) for 10 min. After cooling and a 30-min incubation period at room temperature, the samples’ absorbance was recorded at a wavelength of 620 nm. Carbohydrate quantification was performed using a calibration curve with glucose as the reference standard. The carbohydrate content was calculated based on the glucose calibration curve.

#### 2.4.6. Lipid Determination

Lipid quantification was performed using a spectrophotometric method based on lipid degradation in the presence of a phosphovanillin reagent [31]. The lipid extraction procedure involved treating 10 mg of biomass with 1.0 mL of a chloroform–ethanol (Dita EstFarm SRL, Cojusna, Republic of Moldova) mixture in a 9:1 volumetric ratio. The resulting extract was subjected to separation and drying, and the precipitate was hydrolyzed in an acidic medium by adding 1 mL of sulfuric acid and heating for 10 min. After cooling, a 0.1 mL aliquot of the hydrolysate was taken and reacted with 2.9 mL phosphovanillin reagent (1.2 mg of vanillin in 1.0 mL of 68% phosphoric acid). Spectrophotometric determination was performed after 30 min by measuring the absorbance at a wavelength of 520 nm. Lipid content was calculated using a calibration curve prepared with pure oleic acid.

#### 2.4.7. Malondialdehyde (MDA) Determination

Malondialdehyde (MDA) content was determined spectrophotometrically by forming thiobarbituric acid (TBA)-reactive products. The experimental protocol involved adding 3 mL of 0.67% TBA solution in 20% trichloroacetic acid (TCA) to 1 mL of the biomass sample at a 10 mg/mL concentration. The mixture was heat-treated at 100 °C for 20 min, followed by cooling and centrifugation at 3000× *g* for 15 min. The MDA concentration was quantified by measuring the absorbance at 535 nm, corresponding to the MDA-TBA complex’s maximum absorbance. Non-specific pigmentation was corrected by measuring the absorbance at 600 nm. The MDA content was expressed in µg/g of biomass using the molar extinction coefficient specific to the MDA-TBA complex (ε = 1.56 × 10^5^ M^−1^cm^−1^).

### 2.5. Statistical Analysis

The experiments were conducted in three independent replicates. The experimental results were expressed as the mean ± standard deviation (SD). A Student’s *t*-test was used to identify differences between control and experimental conditions. A statistical significance level of *p* < 0.05 was considered relevant. All statistical analyses were performed using Microsoft Excel 2019, version MSO 16.0.10406.20006.

## 3. Results

### 3.1. Biomass Accumulation

**Figure 1:**
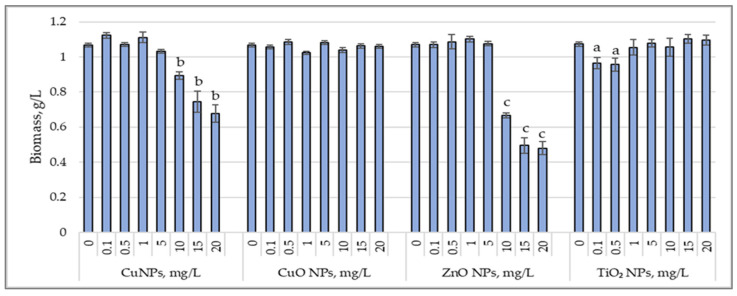
Changes in biomass content (g/L) accumulated by cyanobacterial strain *Arthrospira platensis* CNMN-CB-02 under influence of CuNPs, CuONPs, ZnONPs, and TiO_2_NPs; (0—control, n = 3); a—*p* < 0.05; b—*p* < 0.01; c—*p* < 0.001.

Figure 1 illustrates the variations in biomass accumulation by the cyanobacterium *Arthrospira platensis* under the influence of CuNPs, CuONPs, ZnONPs, and TiO_2_NPs.

Applying CuNPs at concentrations of 0.1 to 5.0 mg/L did not affect biomass production, with the final biomass content being similar to that of the control sample. High concentrations of CuNPs, ranging from 10 to 20 mg/L, reduced biomass content by 15.6% (*p* < 0.05) to 37.4% (*p* < 0.01). CuONPs, at the applied concentrations, did not alter biomass production, which remained at the level of the control sample. For ZnONPs, high concentrations of 10 to 20 mg/L caused a significant decrease in the final biomass content, ranging from 37.7% (*p* < 0.001) to 55.8% (*p* < 0.001). TiO_2_NPs did not exhibit an inhibitory effect on biomass production by *Arthrospira platensis* at most applied concentrations, except for 0.1 mg/L and 0.5 mg/L, which reduced biomass content by 9.7% (*p* < 0.05) and 10.6% (*p* < 0.05), respectively.

### 3.2. Protein and Carbohydrate Accumulation

**Figure 2:**
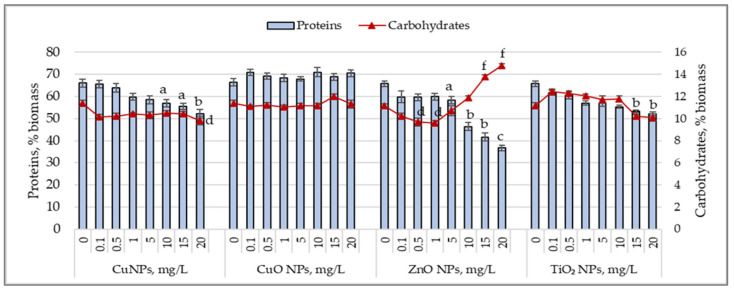
Changes in the protein content (%) and carbohydrate content (%) of *Arthrospira platensis* CNMN-CB-02 biomass cultivated in the presence of CuNPs, CuONPs, ZnONPs, and TiO_2_NPs; (0—control, n = 3); a—*p* < 0.05; b—*p* < 0.01; c—*p* < 0.001 for protein content; d—*p* < 0.05; f—*p* < 0.001 for carbohydrate content.

Figure 2 shows the changes in protein and carbohydrate content in *Arthrospira platensis* biomass cultivated under the influence of the tested nanoparticles.

Lower concentrations of CuNPs (0.1–0.5 mg/L) did not influence protein synthesis, but higher concentrations caused a decrease in protein content in cyanobacterial biomass, ranging from 9.4% (*p* < 0.05) to 20.9% (*p* < 0.01). The carbohydrate content decreased by 8.2% at the concentration of 10 mg/L and by 13.7% at 20 mg/L of CuNPs. CuONPs, at all tested concentrations, did not cause significant variations in the protein or carbohydrate content of the *Arthrospira platensis* biomass. Adding ZnO nanoparticles to the culture medium at concentrations of 0.1–1.0 mg/L reduced the protein content by 10% and the carbohydrate content by 8.3–13% (*p* < 0.05). At concentrations between 5 and 20 mg/L, ZnONPs induced a decrease in the protein content ranging from 11.3% (*p* < 0.05) to 44.2% (*p* < 0.01) compared to the control, while the carbohydrate content increased by 23.6–32.5% at 15 mg/L and 20 mg/L of ZnONPs. TiO_2_ nanoparticles, at concentrations of 1–20 mg/L, reduced the protein content in cyanobacterial biomass by 13.3% (*p* < 0.05) to 29.8% (*p* < 0.01), while fluctuations in the carbohydrate content were insignificant.

### 3.3. Phycobiliprotein Accumulation

**Figure 3:**
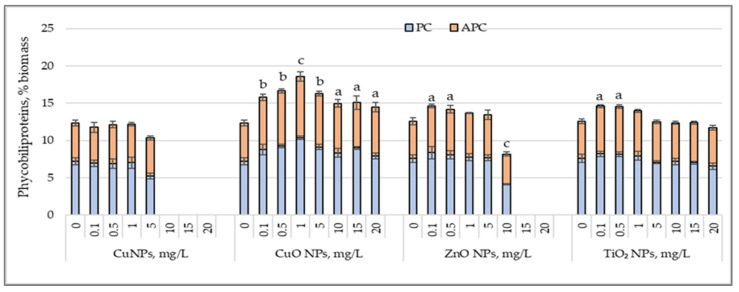
Changes in the phycobiliprotein content (%) in the biomass of *Arthrospira platensis* CNMN-CB-02 cultivated in the presence of CuNPs, CuONPs, ZnONPs, and TiO_2_NPs; (0—control, n = 3); a—*p* < 0.05; b—*p* < 0.01; c—*p* < 0.001.

Figure 3 illustrates the distinct responses of phycobiliproteins in *Arthrospira platensis* biomass cultivated in the presence of CuNPs, CuONPs, ZnONPs, and TiO_2_NPs.

Low concentrations of CuNPs (0.1–1.0 mg/L) did not significantly affect phycobiliprotein synthesis; so, the total phycobiliprotein content remained close to that of the control sample. However, at a concentration of 5 mg/L, there was a reduction in phycobiliproteins by 16.04% (*p* < 0.05), primarily due to the decrease in phycocyanin levels, which showed a significant decrease of 27.6% (*p* < 0.01). At higher concentrations of CuNPs, between 10 and 20 mg/L, phycobiliprotein synthesis was significantly inhibited, with *Arthrospira platensis* biomass devoid of auxiliary pigments. At concentrations ranging from 0.1 to 5.0 mg/L, the total phycobiliprotein content increased by 28.07% (*p* < 0.01) to 50.5% (*p* < 0.001) compared to the control. This increase resulted from the enhanced synthesis of both phycocyanin and allophycocyanin. At higher CuONP concentrations, between 10 and 20 mg/L, the total phycobiliprotein content increased less markedly, reaching a rise of 17.4% (*p* < 0.05) to 22.2% (*p* < 0.05). This amplification of synthesis was mainly attributed to allophycocyanin, which recorded significantly higher values, with increases ranging from 18.9% (*p* < 0.01) to 29.6% (*p* < 0.001). Zinc oxide nanoparticles, at low concentrations of 0.1 and 0.5 mg/L, led to a moderate increase in phycobiliprotein content, with values higher by 16.4% (*p* < 0.05) and 12.7% (*p* < 0.05), respectively, compared to the control sample. The increase occurred mainly due to allophycocyanin, which increased by 26% (*p* < 0.01) and 18.7% (*p* < 0.05), respectively, compared to the control sample. However, at a concentration of 10 mg/L of ZnONPs, a significant decrease in the total phycobiliprotein content was observed, reducing by 35% (*p* < 0.001), mainly due to the decrease in phycocyanin levels of 45.5% (*p* < 0.01), demonstrating the inhibitory effect of ZnONPs at this concentration. TiO_2_ nanoparticles, applied in low concentrations of 0.1 and 0.5 mg/L, also positively influenced phycobiliprotein content, causing an increase of 16.1% (*p* < 0.05) and 15.4% (*p* < 0.05), respectively, compared to the control. These increases were due to higher values of allophycocyanin, which showed an increase of 26.4% (*p* < 0.01)–27.4% (*p* < 0.01) compared to the control. At the other tested TiO_2_ concentrations, no significant variations in phycobiliprotein content were observed, indicating a limitation of the stimulative effect of TiO_2_ at very low concentrations.

### 3.4. Chlorophyll and Carotenoid Accumulation

**Figure 4:**
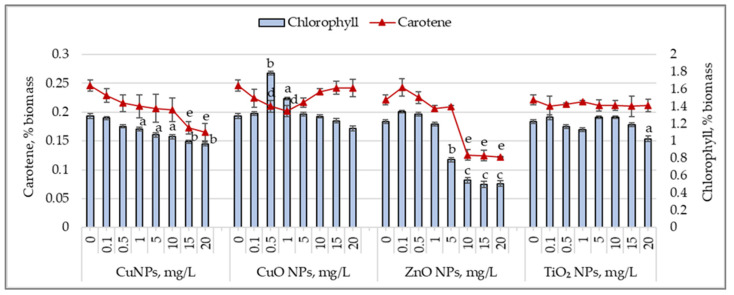
Changes in the chlorophyll content (%) and carotene content (%) in *Arthrospira platensis* CNMN-CB-02 biomass cultivated in the presence of CuNPs, CuONPs, ZnONPs, and TiO_2_NPs; (0—control, n = 3); a—*p* < 0.05; b—*p* < 0.01; c—*p* < 0.001 for chlorophyll content; d—*p* < 0.05; and e—*p* < 0.01 for carotene content.

Figure 4 shows the changes in the chlorophyll and carotenoid content in *Arthrospira platensis* biomass following exposure to CuNPs, CuONPs, ZnONPs, and TiO_2_NPs during cultivation.

CuNPs caused a reduction in chlorophyll content, ranging from 11.9% (*p* < 0.05) to 25.2% (*p* < 0.01), starting at a concentration of 1.0 mg/L. The carotenoid content decreased more prominently. A concentration of 0.5 mg/L CuNPs reduced the carotenoid level by 12.3%, while 20 mg/L caused a decrease of 32.8% (*p* < 0.01) compared to the control sample. CuONPs had different effects on pigments. Concentrations of 0.5 and 1.0 mg/L increased the chlorophyll content by 38.4% (*p* < 0.01) and 15.5% (*p* < 0.05), respectively, while the carotenoid content decreased by 14.4% (*p* < 0.05)–17.9% (*p* < 0.05). At 20 mg/L, the chlorophyll content decreased by 11.3%, while the carotenoid content remained unchanged. Other concentrations of CuONPs did not significantly affect pigment levels in *Arthrospira platensis* biomass. For ZnONPs, low concentrations (0.1–1.0 mg/L) did not significantly affect chlorophyll and carotenoid content. However, higher concentrations caused a reduction in the chlorophyll content by 35.9% (*p* < 0.05) to 58.5% (*p* < 0.001). The carotenoid content decreased by 43.2% (*p* < 0.01) to 44.8% (*p* < 0.01) in samples cultivated with ZnONPs at 5.0–20.0 mg/L concentrations. TiO_2_ nanoparticles, at all tested concentrations, did not affect the chlorophyll or carotenoid content in *Arthropsira platensis* biomass, except for the 20 mg/L concentration, which decreased the chlorophyll content by 16.1% (*p* < 0.05)

### 3.5. Lipid Content and Malondialdehyde (MDA) Accumulation

**Figure 5:**
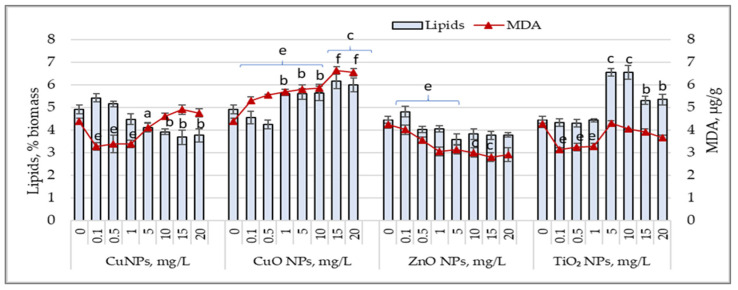
Changes in the lipid content (%) and MDA values (µg/g) in *Arthrospira platensis* CNMN-CB-02 biomass cultivated in the presence of CuNPs, CuONPs, ZnONPs, and TiO_2_NPs; (0—control, n = 3); a—*p* < 0.05; b—*p* < 0.01; c—*p* < 0.001 for lipid content; e—*p* < 0.01; f—*p* < 0.001 for MDA values.

Figure 5 shows the variations in lipid content and malondialdehyde (MDA) accumulated in biomass following the exposure of cyanobacterial culture to the action of the studied nanoparticles. CuNP concentrations between 5 and 20 mg/L reduced the lipid content in biomass by 16.3% (*p* < 0.05) to 23.6% (*p* < 0.01), with an insignificant increase in MDA levels.

Low concentrations of CuNPs resulted in MDA values that were 22.9% (*p* < 0.01) to 25.3% (*p* < 0.01) lower than those of the control sample, while lipid content showed no significant variations. CuONPs, at all applied concentrations, caused a significant increase in MDA levels by 21% (*p* < 0.01) to 51.5% (*p* < 0.001) above the control level. Lipid content increased by 21.9% (*p* < 0.01) to 25.2% (*p* < 0.001), except at concentrations of 0.1 and 0.5 mg/L, which caused a reduction of 7.3% to 13.8% in lipids in *Arthrospira platensis* biomass. ZnONPs in all applied concentrations significantly reduced MDA levels in cyanobacterial biomass, with values 26.3% (*p* < 0.01) to 34.2% (*p* < 0.01) lower than the control. At concentrations of 15 mg/L and 20 mg/L, MDA values decreased by over 100%. In most experimental samples, the lipid content in *Arthrospira platensis* biomass decreased by 9.6% to 19.6%. For TiO_2_NPs, low concentrations led to a reduction in MDA values by 23% to 26.3%, while lipid content remained unchanged. TiO_2_NP concentrations between 5 mg/L and 20 mg/L increased lipid content by 19.5% (*p* < 0.05) to 47.6% (*p* < 0.01) above the control value, while the MDA levels in these samples remained unchanged or were reduced.

## 4. Discussion

The interaction between nanoparticles and aquatic microorganisms varies depending on the type and concentration of nanoparticles. Most studies analyze the response of microalgae and cyanobacteria to nanoparticles based on their toxic effects, which depend on the type and concentration of nanoparticles.

The factor that triggers the cascade of changes in microalgal cells is the generation of reactive oxygen species (ROS). These are produced through various mechanisms influenced by the presence of nanoparticles in the cellular environment. Nanoparticles can interact directly with the cell membranes of microalgae, disrupting their structure and function. These interactions may release soluble metal ions or other reactive chemicals, generating ROS [17]. Nanoparticles can also affect mitochondrial function, as mitochondria are cells’ primary source of ROS. Such interactions may disrupt the mitochondrial respiratory chain [3].

Additionally, nanoparticles may negatively impact photosynthesis in microalgae by impairing photosystem II (PSII) and chlorophyll content. Semiconductor nanoparticles, such as TiO_2_ or ZnO, can generate radicals through photocatalysis [32]. Nanoparticles may agglomerate within cells, forming large complexes that could interfere with normal cell functions, interact with biomolecules, or limit access to light [26].

These mechanisms can result in an imbalance between ROS production and the capacity of the microalgal cells’ antioxidant systems to neutralize it. Consequently, oxidative damage to lipids, proteins, and cellular DNA may occur. This damage can adversely affect microalgal growth and survival and, in severe cases, lead to cell death.

Numerous studies have documented the toxicity of CuNPs and ZnONPs, showing that the toxic effects of CuNPs vary depending on the microalgal species studied [23,33]. The analysis of the impact of copper and zinc oxide nanoparticles on the cyanobacterium *Arthrospira platensis* revealed a common toxicity model at high concentrations of 10–20 mg/L. Both types of nanoparticles caused a significant reduction in biomass and substantial changes in the biochemical composition of the culture.

A notable effect was the drastic reduction in photosynthetic pigments and the complete disappearance of phycobiliproteins. A decrease in protein and lipid content accompanied these changes. The reduction in photosynthetic pigments and the absence of phycobiliproteins confirm the toxicity of CuNPs and ZnONPs toward *Arthrospira platensis*. Additionally, the decrease in lipid content suggests the reduced ability of the cells to cope with oxidative stress, which is summarized by the increased rigidity of the membranes (Liang, 2020) [3]. Some authors consider the reduction in phycobiliprotein content to be an adaptive response to the action of ZnONPs. The binding of Zn ions by phycobiliproteins limits nanoparticle absorption and reduces toxicity [34]. However, the effects of action differed significantly between copper and zinc oxide nanoparticles.

In this study, increased MDA levels were observed only with CuONPs, while ZnONPs led to significantly lower MDA levels. Although a low lipid content is typically associated with reduced MDA levels, this was not true for CuNPs. The lipid quantity reduction caused by CuNPs was similar to that caused by ZnONPs. In this context, MDA levels, in the absence of correlation with other parameters, do not unambiguously reflect the toxicity of the nanoparticles on *Arthrospira platensis*.

These results suggest that ZnONPs may strongly inhibit biosynthetic activity, significantly reducing the functional components of the biomass. The reduced biosynthetic activity caused by high concentrations of ZnONPs limits the excessive accumulation of free radicals, preventing high MDA levels. A 20–30% increase in carbohydrate content could be a condition for maintaining the viability of *Arthrospira platensis* in these conditions.

Biosynthetic activity appears to be redirected toward carbohydrate accumulation as a carbon reserve, even though high concentrations of ZnONPs affect lipid synthesis. In contrast, CuNPs did not significantly inhibit the biosynthetic activity of the cyanobacterium. The structural and functional components of *Arthrospira platensis* remained at relatively low levels. Carbohydrate content was unaffected, and in conditions of active biosynthetic activity and high concentrations of nanoparticles, the excessive accumulation of MDA indicates oxidative stress within the cells.

At low CuNP and ZnONP concentrations, the biochemical composition of *Arthrospira platensis* biomass showed no significant changes and the biomass content remained stable. However, both types of nanoparticles influenced protein synthesis. Protein content decreased proportionally with nanoparticle concentration, showing a strong negative correlation: r^2^ = −0.797 for CuNPs and r^2^ = −0.943 for ZnONPs.

Similar results were observed in the CuNP toxicity tests, with an EC50 of 0.991 mg/L for *Skeletonema costatum* and 2.455 mg/L for *Nitzschia closterium*. These doses are moderately toxic to *Arthrospira platensis* [23]. Copper exposure is known to reduce microalgal growth rates in a dose-dependent manner. High copper concentrations decrease cell density and division rates, impacting chloroplasts and photosynthetic efficiency [35].

In this study, CuNPs induced oxidative stress in cells, as evidenced by the reduced chlorophyll and carotenoid levels. However, the stress remained moderate, indicated by the high lipid levels and low MDA values.

At low concentrations, ZnONPs stimulated the synthesis of phycobiliproteins in the culture, along with increased levels of chlorophyll and carotenoids. This suggests either a stimulatory effect of ZnONPs or an adaptive mechanism of the culture to maintain antioxidant balance, mainly as MDA levels were significantly lower than in the control. At these low concentrations, *Arthrospira platensis* appears to exhibit an adaptive response by stimulating the synthesis of photosynthetic pigments and maintaining low MDA and protein levels in a stable balance. Some authors consider that such changes, including activating antioxidant responses, enhancing the quantity of photosynthetic pigments, and adjusting cellular transport mechanisms, demonstrate the microalgae’s adaptation to low nanoparticle concentrations, supporting growth and metabolic activity [36].

In a study on the short-term effects of CuNPs on the marine diatom *Phaeodactylum tricornutum*, concentrations below 40 mg/L induced slight growth, suggesting a possible hormetic effect [37]. A similar response was observed with metal ions, such as *cadmium*, where low concentrations induced a hormetic effect in *Chromochloris zofingiensis*, enhancing photosynthetic processes without increasing MDA levels, thereby contributing to the maintenance of cellular redox balance [38].

At high doses of nanomaterials, the hormetic effect is reversed, leading to inhibited growth and metabolic activity. Toxicity at high nanomaterial concentrations manifests through impaired photosynthetic processes, strong oxidative stress, reactive oxygen species accumulation, and cellular structural damage [36]. For example, a concentration of 10 mg/L of ZnONPs significantly reduced chlorophyll content, negatively affecting photosynthesis and growth in *Chlorella* sp. [39]. In the case of *Scenedesmus rubescens*, the long-term toxicity of ZnONPs was studied, revealing that concentrations above 8.1 mg/L significantly inhibited microalgal growth [40]. These toxic concentrations are consistent with those determined in the present study.

The experiments showed that concentrations above 10 mg/L of ZnONPs inhibited phycobiliprotein synthesis. At a concentration of 50 mg/L, ZnONPs reduced phycocyanin by 61.8%, accompanied by a 75% decrease in chlorophyll and a 64.1% reduction in carotenoids in *Spirulina platensis* [41]. Another study on *Chlorococcum* sp. investigated the short-term effects of varying ZnONP concentrations (ranging from 0.081 to 810 mg/L) on growth rates and nutrient absorption. At high concentrations exceeding 8.1 mg/L, growth and nitrate uptake decreased significantly by up to 40.1% compared to the control. The toxicity of ZnONPs was attributed to oxidative stress, which affected cell membrane permeability, photosynthesis, and nutrient absorption [42]. Consequently, the disappearance of phycobiliproteins in both experimental conditions could result from thylakoid membrane damage and the inability of the culture to regenerate them.

The inhibition of *Arthrospira platensis* biosynthetic activity by high ZnONP concentrations could be caused by interference with cellular transport and nutrient utilization, limiting the resources needed for biomolecule synthesis. Nanoparticles may influence the expression of genes involved in biosynthesis or inhibit the activity of essential enzymes, thereby reducing the efficiency of metabolic processes without causing significant radical accumulation [36]. Mechanical effects, such as nanoparticle aggregation on the cell surface, can create a physical barrier that hinders gas exchange and nutrient absorption, contributing to biosynthesis inhibition without inducing intense oxidative stress [39,43].

The identified mechanisms of ZnONP toxicity include physical damage to cell membranes, the reduction in photosynthetic pigment levels, and the inhibition of nutrient absorption [41].

This study revealed a similar response of *Arthrospira platensis* cultures to high nanoparticle concentrations, characterized by reduced biomass and changes in biochemical composition. However, the mechanisms of action differ between copper and zinc oxide nanoparticles, as evidenced by adaptive reactions at low concentrations, where cultures develop specific mechanisms for each nanoparticle type. Thus, each type of nanoparticle generates a unique response, even though the overall effect on the culture at high doses is similar.

The interaction of *Arthrospira platensis* with copper oxide and titanium dioxide nanoparticles produced distinct responses. Exposure to TiO_2_NPs maintained biomass content at levels typical for cultures grown under laboratory conditions. However, reduced protein levels suggest the potential toxicity of TiO_2_NPs, even though photosynthetic pigments remained unaffected. Furthermore, the reduction in protein content did not hinder phycobiliprotein synthesis, which was stimulated at low nanoparticle doses. At high doses, lipid content was increased, and most tested concentrations induced carbohydrate accumulation.

A similar lipid synthesis stimulation effect was reported for *Chlorella vulgaris* at TiO_2_ concentrations below 1.0 g/L for short exposure periods as an adaptive mechanism to stress through the simultaneous accumulation and degradation of fatty acids [5]. Additionally, increased MDA levels are a marker of TiO_2_NPs toxicity, indicating induced oxidative stress [44]. For these nanoparticles, toxicity has been associated with damage to photosynthetic pigments at concentrations up to 1 mg/L, as also observed in the present study. At concentrations above 5 mg/L, the photosynthetic system of *Chlorella vulgaris* is severely affected. Reduced protein content was noted in *Phaeodactylum tricornutum* at concentrations exceeding 10 mg/L, while concentrations above 5 mg/L stimulated lipid content as a mechanism to preserve membrane integrity in *Raphidocelis subcapitata* and *Phaeodactylum tricornutum* [25].

Thus, the mechanisms for inducing toxicity and maintaining viability are similar in the presence of TiO_2_NPs, as observed in our study with *A. platensis*. The culture adapted through well-documented mechanisms, such as increased lipid and phycobiliprotein content, which exhibit antioxidant effects. Low MDA levels indicate that this cyanobacterial culture effectively copes with induced oxidative stress.

The results obtained from cultivating the cyanobacterium *Arthrospira platensis* in the presence of copper oxide nanoparticles are remarkable. Cyanobacterial growth was unaffected, and the culture exhibited a slight increase in protein content, remaining within the typical range for this strain. Moreover, a significant increase in phycobiliproteins was observed. Oxidative stress effects were noted only at high CuONP concentrations, reflected in an increase in lipid content. MDA levels were elevated across all tested concentrations, indicating a concentration-dependent oxidative stress response with a strong positive correlation (r^2^ = 0.9367).

Generally, the microalgal response to CuONPs involves activating dose-dependent oxidative stress mechanisms [18]. Some studies suggest that microalgal tolerance to CuONPs may be linked to adaptive mechanisms, including the production of ligands and extracellular polymers that protect against copper toxicity [22]. For example, exposure to CuONPs at concentrations of 3000, 2500, and 1000 µg/L in *Dunaliella salina*, *Isochrysis galbana*, *Thalassiosira weissflogii*, and *Prorocentrum cordatum* induced severe oxidative stress. However, the photosynthetic systems of these species showed a high functional tolerance to CuONPs [45]. In our research, *Arthrospira platensis* demonstrated photosynthetic stability despite elevated MDA levels.

The increase in phycobiliprotein content in response to CuONPs suggests their potential role in maintaining the culture’s redox balance. Elevated phycocyanin levels, known for their antioxidant effects, support this hypothesis.

This study identified various interactions between the cyanobacterium *Arthrospira platensis* and different toxic nanoparticles, with evaluations conducted at the end of the cultivation cycle. Copper nanoparticles exhibited a pronounced toxic effect on A. platensis, characterized by reduced biomass, protein content, and photosynthetic pigments, in a concentration-dependent manner. In contrast, low concentrations of ZnONPs displayed a hormetic effect, stimulating biosynthetic activity at low doses. Titanium dioxide nanoparticles had a relatively neutral effect, with moderate oxidative stress symptoms, while copper oxide nanoparticles stimulated biosynthetic activity across all tested concentrations.

The toxicity indicators were represented by biomass content and photosynthetic pigment reductions, including the lack of phycobiliproteins. For CuNPs, toxicity is evident. As an adaptation mechanism, increased lipid and carbohydrate content was observed. Additionally, the synthesis of phycobiliproteins was stimulated at low doses of ZnONPs and TiO_2_NPs. For CuONPs, it can be argued that this stimulation of phycobiliprotein synthesis is more likely an adaptive response rather than one strictly aimed at maintaining the redox status.

The increase in MDA levels suggests that cells are subjected to significant oxidative stress. The synthesis of phycobiliproteins may represent a compensatory biosynthetic response, not necessarily aiming to maintain the cell’s redox status but as part of protective and adaptive mechanisms to oxidative stress.

In conclusion, this increase in phycobiliprotein synthesis may reflect a compensatory response to toxicity rather than a mechanism to maintain homeostasis. Furthermore, MDA levels should be considered indicators of oxidative stress and markers of the intensity of biosynthetic processes within the cell.

The experiments established the effects of the interaction of Cu, CuO, ZnO, and TiO_2_ nanoparticles with the cellular structures of the cyanobacterium *A. platensis*. As a result of nanoparticle toxicity, the photosynthetic system was affected, evidenced by a reduction in chlorophyll content, membrane structure damage manifested by increased lipid content and MDA accumulation, and changes in the biomolecule content, reflected by a decrease in protein levels. Among the adaptation mechanisms, hormesis-like effects were observed, including the reduction in lipid content necessary for increasing membrane rigidity, the reduction in phycobilin, and the increase in carbohydrate content, which serves as a carbon reserve.

A gap was highlighted between the toxic mechanisms of the nanoparticles and the results of the cyanobacterium’s adaptation process. First, no increases in MDA levels were observed in the experiments with CuNPs, ZnONPs, and TiO_2_NPs, as lipid content was reduced. Secondly, extreme MDA values were recorded following the interaction of CuONPs with the A. platensis culture. In this case, oxidative stress was induced, but rather as a result of CuONPs’ involvement in the cells’ biosynthetic activity, which suggests the absence of toxicity within the applied concentrations.

This opens up the possibility of using nanoparticles as stimulators in biotechnology to produce bioactive substances, where microalgae and cyanobacteria serve as the raw materials. In the case of *A. platensis*, these include proteins with a broad spectrum of amino acids, phycocyanin, gamma-linolenic acid, and sulfated polysaccharides.

All of the parameters analyzed in this study are specified as criteria for monitoring the impact of nanoparticles on aquatic organisms, focusing on their interaction with microalgae and providing a better understanding of the mechanisms of toxicity and their potential for adaptation (Deniel, 2019) [26]. However, the long-term effects of nanoparticles on aquatic biocenoses depend on the functional state of microorganisms, the type of nanoparticles, and their concentration. The consequences or effects of the interaction must be constantly monitored, and specific regulations are necessary for the use of nanoparticles, even for beneficial purposes.

## 5. Conclusions

The cyanobacterium *Arthrospira platensis* CNMN-CB-02 exhibited different responses to interactions with copper, copper oxide, zinc, and titanium nanoparticles, depending on their type and concentration. At high concentrations (10–20 mg/L), nanoparticle toxicity led to reduced biomass and significant biochemical changes.

High concentrations of CuNPs caused the complete disappearance of phycobiliproteins, reducing the cells’ photosynthetic capacity. Elevated MDA levels in the cyanobacterial biomass indicated severe oxidative stress, characterized by membrane damage and the excessive accumulation of free radicals.

High concentrations of ZnONPs also inhibited phycobilin synthesis. However, the low levels of MDA suggested a limited accumulation of free radicals, likely due to the inhibition of biosynthetic activity. The maintenance of culture viability under these conditions was reflected in the increased carbohydrate content, resulting from the redirection of metabolism toward carbohydrate synthesis as a carbon reserve—a mechanism of adaptation to stress.

TiO_2_NPs and CuONPs induced adaptive effects, manifested by the accumulation of phycobiliproteins. This demonstrates the cyanobacterium’s ability to maintain redox balance and photosynthetic stability even under oxidative stress conditions.

At low concentrations, ZnONPs and TiO_2_NPs exhibited hormetic effects, reflected in the stimulation of photosynthetic pigments, phycobiliproteins, and lipids. These adaptive responses highlight the activation of cellular mechanisms to maintain homeostasis and support growth in the presence of nanoparticles.

MDA levels are not always a reliable indicator of nanoparticle toxicity. For instance, CuNPs caused significant increases in MDA levels, accompanied by a reduction in lipid content, indicating severe and dose-dependent oxidative stress. In contrast, ZnONPs resulted in low levels of lipids and MDA, suggesting general inhibition of biosynthetic processes and reduced reactive oxygen species accumulation.

The elevated MDA levels in the presence of CuONPs and the increased phycobiliprotein content suggest the activation of compensatory mechanisms for oxidative stress. The photosynthetic stability of *Arthrospira platensis* under these conditions reflects its ability to activate efficient protection and adaptation mechanisms.

These findings pave the way for in-depth studies on the molecular mechanisms regulating the adaptive response of *Arthrospira platensis* to nanoparticles. The redirection of biosynthetic activity in *Arthrospira platensis* under stress conditions induced by specific nanoparticles, accompanied by low MDA levels, could be exploited as a biotechnological approach for producing biomolecules of interest.

## Data Availability

The original contributions presented in this study are included in the article; further inquiries can be directed to the corresponding author.
